# Mental health care use in men with comorbid diabetes and depression: The role of age and race

**DOI:** 10.15761/hec.1000163

**Published:** 2019-10-01

**Authors:** Jaclynn M Hawkins, Claudia Schwenzer, Hillary K Hecht, Lenette Jones, Daniel Velez-Ortiz, Jaewon Lee, Brian Ahmedani, Gretchen Piatt

**Affiliations:** 1 School of Social Work University of Michigan, USA; 2 School of Nursing University of Michigan, USA; 3 School of Social Work Michigan State University, USA; 4 Henry Ford Health System, Detroit, MI, USA; 5 School of Medicine, University Michigan, USA

## Abstract

**Objectives:**

Older adults with diabetes have double the normal average risk for depression. While women also report higher rates of depression, men are less likely than women to recognize symptoms and seek assistance for mental health treatment. Racial disparities in mental health care use among men have also been identified. While age and gender differences in mental health care use have been accounted for in adults with comorbid diabetes and depression little is known about within group differences among men. The purpose of this study was to examine the influence of age and race on mental health service use in a sample of men with comorbid diabetes and depression.

**Methods:**

This study utilized secondary data from a large health care delivery system serving in a Midwestern urban city and included 335 Black, and non-Latino White men with comorbid type 2 diabetes and depression.

**Results and Discussion:**

Findings indicate that men under the age of 55 were less likely to experience a 6-month or more delay in receiving a psychiatric medication prescription after their initial depression diagnosis. Black men over 55 years of age were significantly more likely to experience a delay of over six months to receiving psychiatric medication. More research is needed to explore preferred depression treatment methods for older Black men with type 2 diabetes, in addition to any issues with access to pharmacological medications to treat depression.

## Introduction

In the United States, 23.1% of adults 60 years of age and older have diabetes [1]. Diabetes is associated with a significant increase in risk and prevalence for clinical depression and depressive symptoms [2–5] and older adults with diabetes have double the normal average risk for depression [6]. Adults over the age of 60 with diabetes face a number of unique risk factors for depression, such as admittance to extended care facilities and higher incidence of comorbidities. These risk factors contribute to increases in depressive disorders [7]. Further, adults in later life with comorbid depression and diabetes are less likely to adhere to medication, exercise and diet regimens and are more likely to experience reductions in quality of life and increased medical expenditures [8]. Considering the increased risk for depression among older adults with diabetes, the treatment of depression is critical to achieving optimal health [9]. However, mental health status is often overlooked in health care settings as a common comorbidity alongside chronic diseases such as diabetes [10].

Gender differences in diabetes and depression diagnosis also exist in diabetes and depression diagnosis and treatment. Research has shown that women have a slightly higher incidence of diabetes than men, yet, diagnosis rates for men have increased at a faster rate over the last 20 years [11]. Moreover, while women also report higher rates of depression, men are less likely than women to recognize symptoms and seek assistance for mental health treatment [12–14]. Research indicates that men do not receive mental health treatment mainly due to a lack of awareness of symptoms, social stigma, and reluctance to ask for help [12–14]. Given these barriers, men with mental health issues may utilize unhealthy coping strategies such as drinking alcohol, using drugs, or engaging in aggressive behaviors, rather than asking for help [15].

In general, depression is often treated with prescription of psychiatric medication and/or therapy delivered by a mental health professional [16]. Pharmacological and psychotherapeutic interventions have been shown to successfully treat a range of depressive disorders yet, research has shown that women and young adults are more likely to actively seek out and utilize mental health services compared to men [16–19]. Gender disparities in mental health help seeking also remain constant over the life course, with older men more likely to underutilize mental health treatment for their depression than older women [20–22].

Racial disparities in mental health care use among men have been studied elsewhere [23–24]. However, to our knowledge, no studies have been conducted that examine both race and age differences in mental health service use among men with comorbid diabetes and depression. Considering the critical intersection of race, age, and gender in mental health care use, additional attention is needed to identify predictors of mental health seeking among men managing diabetes to highlight racial disparities at these afore mentioned intersections. Therefore the purpose of this study was to examine the influence of age and race on mental health service use in a sample of men with comorbid diabetes and depression. This study investigates this important gap in knowledge, and also extends previous work by examining racial differences in both mental health service use and prescription use of psychiatric medication to treat depression in men with diabetes. Depression can have a damaging impact on diabetes management, therefore, assessing predictors of mental health care use for men with diabetes may translate into strategies to reduce diabetes-related disparities.

## Methods

### Sample

The present study uses de-identified medical record data from a large, integrated delivery and financial system serving the residents of an urban Midwestern city. Medical records were used to identify patient’s demographic information, laboratory testing results (type 2 diabetes diagnosis), depression diagnosis, prescription drug use and visits with a mental health professional. After restricting the sample to men 18 years or older with a diagnosis of type 2 diabetes and those with complete data for the variables used in the study, the sample included 335 Black, and non-Latino White men with comorbid type 2 diabetes and depression.

## Measures

### Outcome variables

Mental health care after first depression diagnosis was defined as either 1) seeing a mental health care professional or 2) receiving psychiatric medication to treat depression. Date of mental health care treatment and prescription of psychiatric medication were abstracted from patient electronic medical records. These variables were measured with two items: first, length of time before *seeing a mental health care professional* after first depression diagnosis was defined as: (0) less than or equal to 6 months, and (1) longer than 6 months. Second, length of time before *receiving psychiatric medication* to treat depression after first depression diagnosis was coded as (0) less than or equal to 6 months and (1) longer than 6 months. These two variables were used to determine the occurrence and timing of first treatment contact after depression was diagnosed. Receipt of treatment within 6 months was chosen due to sample size constraints and the variability in the average time to see a mental health care professional once diagnosed by a primary care physician. For persons living with diabetes, receiving treatment for depression in a timely manner is critical. Medical guidelines show that upon depression diagnosis, for treatment to be effective, it should be received within a timely manner [25].

### Independent variables

Self-reported *Race* was a key independent variable, coded as (0) non-Latino White, and (1) Black. Socio-demographic control measures identified in previous literature as being related to health care use included household *income* was coded as (0) less than or equal to $20,000, and (1) greater than $20,000*, education* was coded as (0) less than high school, (1) high school degree or GED, and (2) more than high school; *age* was coded as (0) less than 55, and (1) 55 or older. Lastly, *health insurance status* was coded as (0) public or (1) private.

### Data analysis

Demographic characteristics are presented for this sample of men; to compare sociodemographic characteristics across racial groups. A t-test was used for continuous variables, Fisher’s exact test was used for categorical variables with expected count under 5 in any cell, and Pearson’s chi-square test was used for all other categorical variables. Multiple logistic regression models were used to identify predictors of delayed mental health care treatment. Model 1 examined the association of race with meeting with a mental health professional. The same analysis was run for model 2 with prescription of psychiatric medication to treat depression as the outcome variable. Each model adjusted for sociodemographic covariates. Research has shown that in general, younger individuals are more likely to receive mental health treatment and older Blacks report a higher prevalence of depressive symptoms when compared to older non-Hispanic Whites [26]. Therefore, a race by age interaction effect was included to test for differences across age and racial background. Statistical analyses were conducted using Stata software version 13 [27]. The present study was exempt from the internal review board process because it used a de-identified secondary data set.

## Results

### Participant characteristics

Table 1 outlines demographic information for the study sample. Blacks (85.6%) were more likely to pursue education after high school graduation compared Non-Hispanic Whites (81.2%) in the sample. Most of Non-Hispanic Whites (98.4%) earned over $20,000 annually, while 11.4% of Blacks reported an annual income of less than $20,000. Conversely, Blacks were more likely to have private insurance in comparison with Non-Hispanic Whites. Both racial groups tend to visit mental health professionals after 6 months after they are diagnosed with depression rather than within 6 months of receiving the diagnosis. Interestingly, after receiving a depression diagnosis, most Non-Hispanic White, and Black men in the sample were more likely to get prescribed a depression medication within 6 months of their depression diagnosis.

### Association between race and mental health treatment

Table 2 reports results for models with the race by age interaction term, adjusting for covariates. Two models were run: Seeing a mental health service provider served as the outcome variable for the first adjusted regression model, while prescription of psychiatric medication was the outcome variable in the second model. Multivariate logistic regression results showed that, for both outcomes, there were no racial differences in predicting seeing a mental health care provider, where the delay extends beyond six months. Analysis also found that men under the age of 55 were less likely to experience a 6 month or longer delay in receiving a psychiatric medication prescription after their initial depression diagnosis (OR = 0.36, 95% CI, 0.18–0.75; p< .01).

### Interaction effects between race and age

Interaction effects were found between age and race predicting the delay of time to the first medication. Older Black men (over 55 years) were significantly more likely (OR = 2.18, 95% CI, 1.12–4.12; p< .05) to experience a delay of over six months to receiving mental health treatment in the form of medication. That is, compared with older non-Hispanic White men in the sample, Black men over the age of 55 were 2.18 times more likely to have a delay of the time to the first medication after a diagnosis of depression where the delay period was longer than six months. Significant race by age differences were not found for experiencing a delay in seeing a mental health professional after a depression diagnosis.

## Discussion

Our study found that for the entire sample, men with type 2 diabetes under the age of 55 were less likely to be delayed (by six months or more) in receiving a psychiatric medication prescription after their initial depression diagnosis. When interaction effects were added to our model, older Black men (55 years+) with type 2 diabetes were significantly more likely to experience a delay of over six months in receiving mental health treatment in the form of psychiatric medication compared to their non-Hispanic white counterparts. This seems to support previous studies indicating that Black adults prefer psychotherapy over prescription drugs for mental health treatment [28–31]. Black men’s potential disinclination to take prescription medications for mental health treatment may result from a complex set of socio-cultural factors such as stigma around taking medication, medical mistrust, perceived clinician bias, and lack of social support around emotional issues [32,33]. Further research is needed to explore Black men’s perceptions about types of preferred treatment for depression and barriers to medication adherence. Both gender and racial differences also exist with regard to preferences for the treatment of depression. In general, Blacks are less likely to utilize mental health services compared to non-Hispanic whites [23,34,35].

Our findings may also partially be explained by previous work showing that Black men with depression seek mental health care less often than White men [36,37] and Black men with major depressive disorders are less likely to use outpatient mental health services compared with Black women [37]. With regard to preferred types of mental health services, one study demonstrated that Black men with mental health needs preferred to meet psychiatrists and non-psychiatrists, such as social workers, psychologists, and counselors rather than primary care physicians in comparison with their female counterparts [23]. In comparison, Black women with mental health needs tend to seek out general medical care and service, such as general physicians, physician specialists, and occupational therapists [23].

Further, older Black may also be less likely to receive psychiatric medication post depression diagnosis because, in general, racial and ethnic minorities are more likely to face various barriers in accessing mental health services resulting in delayed care [38–40]. These barriers stem from a number of multi-level factors. At an individual level, racial/ethnic minorities are more likely to live at or below the poverty level and more frequently have limited health insurance coverage that does not provide access to mental health services; adequate family support to access mental health treatment may also be lacking [41]. On a macro-level, poor community awareness of mental health problems, insufficient funding for mental health care, and a lack of culturally competent, trained staff increase disparities in mental health care treatment for minority populations [41].

## Limitations

As with any study, our findings must be interpreted within a few, but notable limitations. The non-representativeness of the sample limits the ability to generalize our findings. If possible, larger samples of Black men within nationally representative studies and more adequate comparison groups could allow for a closer examination intra-group differences in mental health treatment among men with diabetes. These limitations notwithstanding, this study addresses a gap in understanding gendered and racial disparities in mental health treatment among men with a common medical condition and provides a basis and rationale for additional investigation into the needs of Black men.

## Conclusion

Our findings align with existing literature showing that older Black men face barriers to accessing mental health services [32,42,43] and are less likely to initially seek out mental health treatment [23]. These findings help to extend the literature by adding a more nuanced understanding of factors that contribute to mental health care use, specifically in older Black men with comorbid diabetes and depression, a population that to our knowledge has not been examined in previous work. Our study can be used as a springboard for future studies to more precisely target men with the highest risk for depression and comorbid chronic illness.

## Figures and Tables

**Table 1.** Sample characteristics

Variable	African American (n=90)	Non-Hispanic White (n=245)
**Age**		
Greater than or equal to 55	67.8 (61)	78.4 (192)
Less than 55	32.2 (29)	21.6 (53)
**Education**		
Less than High School	2.2 (2)	2.9 (7)
High School Degree/GED	12.2 (11)	15.9 (89)
More than High School	85.6 (77)	81.2 (199)
**Income**		
Greater than 20K	88.6 (78)	98.4 (239)
Less than or Equal to 20K	11.4 (12)	1.6 (6)
**Insurance**		
Private	55.6 (50)	47.8 (117)
Public	44.4 (40)	52.2 (128)
**No. Days to Mental Health Visit After Depression Diagnosis**		
≤ 6m	6.7 (6)	5.3 (13)
>6m	21.1 (19)	20.8 (51)
No visit	72.2 (65)	73.9 (181)
**No. Days to 1**^**st**^ **Psych. Medical Prescription After Depression Diagnosis**		
≤ 6m	87.7 (79)	93.5 (229)
>6m	12.2 (11)	6.5 (16)

**Table 2.** Odds Ratios from Logistic Regression Models: Time to First Mental Health Visit or Depression Medication Prescription After Depression Diagnosis for Men Age 18 and Older (n=335)

	First Mental Health Visit	First Depression Medication Rx
	OR (95% CI)	OR (95% CI)
**Race**		
Black	0.71 (0.19, 2.65)	0.69 (0.26, 1.83)
Non-Hispanic White (reference)		
**Education**		
More than High School	0.46 (0.08, 2.63)	0.75 (0.39, 1.47)
High School Degree/GED or Less (reference)		
**Age**		
Less than 55	1.97 (0.58, 6.70)	0.36** (0.18, 0.75)
Greater than or equal to 55 (reference)		
**Income**		
Greater than 20K	1.07 (0.28, 4.07)	1.10 (0.62, 1.95)
Less than or Equal to 20K		
**Health Insurance**		
Private	0.55 (0.15, 2.03)	0.71 (0.41, 1.24)
Public (reference)		
**Race** * **Age**		
Black * Greater than or equal to 55	1.15 (0.43, 3.04)	2.18* (1.12, 4.26)

* no delay in mental health care defined as ≤ 6 months and delay defined as >6 months

* P<.05

** P<.01

*** <.001

Abbreviation CI: Confidence Interval
